# Development and characterization of monoclonal antibodies specific for bovine IP-10

**DOI:** 10.1186/s13567-025-01602-z

**Published:** 2025-08-14

**Authors:** Hamza Khalid, Michael Coad, Inga Dry, Catherine McGuinnes, Lindsey A. Waddell, Jayne C. Hope, Zhiguang Wu

**Affiliations:** 1 Division of Immunology, The Roslin Institute, University of Edinburgh, Edinburgh, EH25 9RG UK; 2 Animal and Plant Health Agency, Weybridge, Surrey KT15 3NB UK

**Keywords:** IP-10, intracellular staining, hybridoma technology, monoclonal antibody, bovine tuberculosis

## Abstract

Research on chemokines in cattle is hampered by the relative lack of validated reagents. Bovine IP-10 is an important inflammatory chemokine and a promising diagnostic biomarker for an economically important disease (bovine tuberculosis) caused by infection of cattle with *Mycobacterium bovis*. Currently no monoclonal antibodies are available for bovine IP-10. The goal of this study was to generate novel mAbs for detection of bovine IP-10 using hybridoma technology. Five mAbs were developed and cross clone inhibition analyses showed a high degree of self-inhibition among the mAbs. One mAb (7C2) was used in conjugation with a commercial polyclonal antibody to develop a sandwich ELISA. Upon testing with recombinant bovine IP-10, this ELISA showed an enhanced linear range compared to the currently available polyclonal antibody-based ELISA. The ELISA using 7C2 was shown to detect native antigen-specific bovine IP-10 in samples from *M. bovis* infected animals. The 7C2 mAb could also detect intracytoplasmic IP-10. These novel mAbs will be useful in elucidating roles for IP-10 in bovine immune studies in health, disease and vaccination contexts.

**Supplementary Information:**

The online version contains supplementary material available at 10.1186/s13567-025-01602-z.

## Introduction

Interferon gamma inducible protein 10 (IP-10), also known as C-X-C chemokine ligand 10 (CXCL10) is a low molecular weight (10 kDa) inflammatory cytokine. IP-10 binds to the receptor CXCR3 to exert its biological functions. CXCR3 is a seven transmembrane G protein coupled receptor, found on the surface of Th1 lymphocytes and NK cells, among other cell types. IP-10 is produced by various cell types including monocytes, T lymphocytes, NK cells, endothelial and stromal cells [1]. IP-10 has major role in driving the migration of immune cells towards the site of inflammation [2]. Other biological functions of IP-10 include, but are not limited to, facilitation of T cell adhesion to endothelial cells, modulating the development and function of T cells, and augmenting NK-cell mediated cytolysis by facilitating the discharge of NK cell cytotoxic granules [3–5].

IP-10 has been widely reported as diagnostic biomarker of high relevance for human tuberculosis (TB) [6–10]. Similarly, detection of IP-10 was shown to have promise when measured alongside IFN-γ for the detection of *Mycobacterium bovis* infection in cattle and buffalo [11–14].

Despite being a crucial and discriminant biomarker there are no monoclonal antibodies (mAbs) available for use in assays to detect bovine IP-10. The current available assays utilise polyclonal antibodies (pAbs); we have previously described their use in ELISA and a user friendly lateral flow assay (LFA) format [11, 15] to detect IP-10 in cattle infected with *M. bovis*, the causative agent of bovine TB (bTB). However, these assays presented respective challenges of high background and an overall reduced signal compared to LFAs developed using mAbs for IFN-γ and IL-2 [11, 15]. We reasoned that the use of mAbs would circumvent these challenges and provide opportunities to produce stable reagents of high quality and quantity enabling further characterisation of IP-10 as a diagnostic marker of bTB and its role in other diseases [16, 17].

Here, we describe the generation of novel mAbs to detect bovIP-10 using hybridoma technology [18]. The ability of these mAbs to detect recombinant and native bovIP-10 from bTB was tested in both ELISA and flow cytometric assays.

## Materials and methods

### Animals and ethical statement

All mice were housed at the Bioresearch & Veterinary Service Facility at the Roslin Institute (BVS-Ros facility) at the University of Edinburgh.

Bovine blood samples for mAb testing were taken by venepuncture from the jugular vein of female cattle aged approximately 6 months. These were housed at the University of Edinburgh’s Langhill Farm. Blood samples for measurement of antigen induced IP-10 were also taken from cattle that were bTB reactors (*n* = 17). These were farm animals that had failed a recent bovine TB surveillance skin test and were re-housed at the Animal and Plant Health Agency (APHA) Weybridge site and were found to be culture positive for *M. bovis*. All experimental protocols were carried out under the authority of a UK Home Office Project Licence under the regulations of the Animals (Scientific Procedures) Act 1986 with approval from The Roslin Institute’s Local Animal Welfare and Ethical Review Board (AWERB) or APHA’s AWERB as appropriate. The results are reported in line with the Animal Research: Reporting of In Vivo Experiments (ARRIVE) Guidelines [19].

Additional bovine blood samples (*n* = 14) were obtained from bTB-free herds in Scotland as part of routine veterinary surveillance. The blood samples were stimulated with purified protein derived from *M. bovis* (PPDb) or left unstimulated for 24 h and processed for collection of whole blood supernatants (WBS) as described previously [11, 15]. WBS were diluted 1 in 5 in assay buffer (0.1% bovine serum albumin (BSA, Sigma-Aldrich) and 0.05% Tween-20 (Merck, Gillingham, UK) in PBS) for testing [20].

### Expression and purification of recombinant bovine IP-10 (rbovIP-10)

The sequence corresponding to mature protein of bovIP-10 (NCBI Reference Sequence: NM_001046551.2) with a BsiWI restriction site at the 5’ end and a NheI restriction site at the 3’ end was synthesised (IDT) and cloned into the expression vector pFUSEN-hG1Fc (InvivoGen, San Diego, USA) using the complementary restriction sites BsiWI and NheI to generate bovIP-10 fused with a human IgG1 Fc tag. The resulting plasmid (pFUSEN-bovIP10) was transformed into *E*. *coli* DH5α chemically competent cells according to the manufacturer’s protocol (Thermo Fisher Scientific, Life technologies LTD, Paisley, UK). A Zeocin resistant transformant was selected and the plasmid was sequenced by Sanger sequencing (LightRun Tube services, Eurofins, Ebersberg, Germany) to confirm the open reading frame (ORF). Plasmids for transfection were prepared using EndoFree plasmid kit (QIAGEN, Manchester, UK). Recombinant Fc-IP-10 was expressed using Expi293™ Expression System Kit (Thermo Fisher Scientific) according to the manufacturer’s protocol and purified by using a HiTrap Protein G HP antibody purification column (Cytiva, Fisher Scientific; Loughborough, UK) as per manufacturer’s instruction. Molecular weight was determined by Sodium Dodecyl Sulphate–Polyacrylamide Gel Electrophoresis (SDS-PAGE). Purified bovIP-10 was assessed for its identity and purity using mass spectrometry (MS) by the Proteomics and Metabolomics Facility at RI, University of Edinburgh.

### Development of anti-bovine IP-10 monoclonal antibodies (mAbs)

#### Immunisation of mice

**Figure 1:**
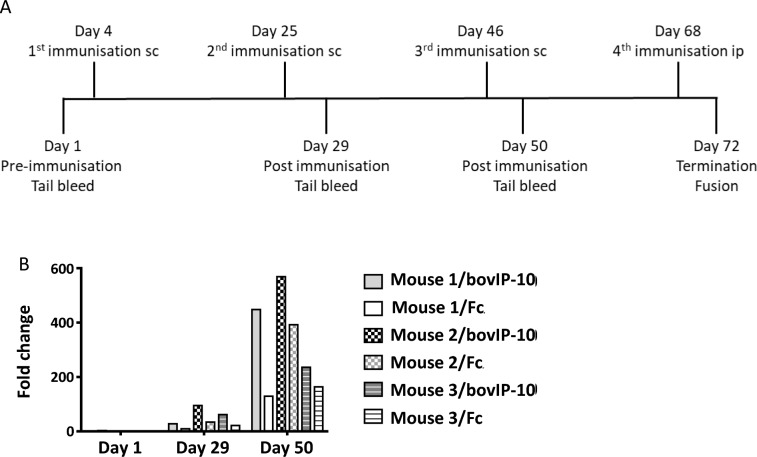
**Immunisation strategy and evaluation of immune response to Fc-bovIP-10 in mice**. **A** Immunisation schemes*.* Three BALB/C strain mice were immunised on day 4, 25 and 46 by subcutaneous injection (sc) with of 50 μg purified Fc-bovIP-10 which was emulsified with TiterMax® Gold Adjuvant (Merck), and on day 68 by intraperitoneal (ip) injection with of 50 μg purified Fc-bovIP-10 without adjuvant. All mice were bled on day 1 (pre-immunisation) and on day 29 and 50 (post-immunisation) from the tail vein to collect blood for sera. Mice were sacrificed for spleens on day 72. **B** The bovIP-10-specific immune responses from mice immunized with recombinant Fc-bovIP-10*.* Following a pre-immunisation test bleed, mice (*n* = 3) were given a total of four immunisations with 50 µg Fc-bovIP-10 protein. Post-immunisation sera were collected following second and third immunisation. Reactivity of the sera (diluted 1 in 1000 in PBS) against Fc-rbovIP-10 and Fc control was tested by indirect ELISA. Fold change refers to the enhanced bovIP-10 reactivity (measured by OD value) of diluted sera compared to the secondary antibody only control well. Averages of two technical replicates were used.

The study design (immunisation of mice, fusion of spleen cells with myeloma cells, hybridomas selection, antibody purification) used here has been described in a recent publication from our laboratory [21]. Three BALB/C strain mice (Charles River Laboratories, UK) were immunized with Fc-bovIP-10 fusion protein for generation of anti-bovIP-10 antibodies. All mice were pre-bled on day 1 (pre-immunisation) from the tail vein to collect blood for serum. On day 4, 25 and 46 each mouse was immunised by subcutaneous injection (sc) of 50 μg purified Fc-bovIP-10 which was emulsified with TiterMax® Gold Adjuvant (Merck). On day 29 and 50 post-immunisation, the mice were bled from the tail vein for serum. Blood was centrifuged at 1000 × *g* for 10 min, serum was collected and stored at −20 °C. A final boost was performed on day 68 by intraperitoneal (ip) injection of 50 μg purified Fc-bovIP-10 without adjuvant. Mice were sacrificed on day 72 for spleen removal. Immunisation scheme is shown in Figure 1A.

### Evaluation of immune response in sera from immunised mice by indirect ELISA

96 well ELISA plates were coated with 50 μL of Fc-IP-10 or human IgG Fc at 1.0 μg/mL, in Carbonate-Bicarbonate Buffer (Sigma-Aldrich) and incubated overnight at 4 °C. The following day, plates were washed with PBS-0.05% Tween-20 (PBST) and blocked using 0.1% Casein (Sigma-Aldrich) in PBS for 1 h at RT. Following three washes with PBST, 50 µL of sera (1/1000 diluted in PBS) from pre- and post-immunisations was added and incubated for 1 h at RT. After a further three washes with PBST, horse anti-mouse IgG-Horseradish-Peroxidase (HRP) secondary antibody (1/5000, Cell Signaling Technology, Dellaertweg, The Netherlands) was added and incubated for 1 h at RT. Following a final three PBST washes, 3,3',5,5'-Tetramethylbenzidine (TMB) substrate (1-Step™ TMB ELISA Substrate Solutions; Thermo Scientific) was used to visualise peroxidase activity. After 10–20 min, 2 M H_2_SO_4_ was used to stop the reaction. Optical density (OD_450_-OD_550_) was measured by a Multiskan Ascent spectrophotometer (BioTek, Winooski, VT, USA) and analysed using Microsoft Excel. Fold change i.e., reactivity of the sera against Fc-rbovIP-10 and Fc control, measured by OD value, relative to the secondary antibody only control wells was calculated. Results for binding of serum antibodies to IP-10 were compared with binding to human Fc.

### Fusion of immunised mouse spleen cells with partner cells, SP2.0/Ag-14

Two days before fusion, mouse thymocytes were grown on flat-bottomed 96-well plates in fusion media [complete media (RPMI-1640 supplemented with 10% HI-FBS, 1 mM Glutamax, 100 U/mL penicillin and 100 µg/mL streptomycin) plus HAT Supplement (Thermo Fisher Scientific); Hybridoma Fusion and Cloning Supplement (HFCS) (Merck); and 2.0 ng/mL recombinant mouse IL-6 (BioLegend, London, UK)] as feeder cells at 37 °C, 5% CO_2_.

Spleens from the three mice were isolated and counted using Trypan Blue exclusion. Prior to counting the cells, cell suspension (100 µL) was mixed with 900 µL of red blood cell lysis buffer (Thermo Fisher Scientific) and incubated for 5 min at room temperature to lyse red blood cells. Cells from mice #1 and #3 were cryopreserved in freezing media [90% FBS/10% DMSO (Merck)] and stored in a −155 °C freezer, while mouse #2 splenocytes were fused with fusion partner Sp2/0-Ag14 mouse myeloma cells [22]. On the day of fusion, Sp2/0-Ag14 cells were harvested, counted and mixed with splenocytes at a ratio of 1:5. The combined cells were centrifuged at 400 × *g* for 5 min. After removal of supernatant, the cell pellet was slowly resuspended in 1 mL pre-warmed Polyethylene glycol solution (PEG) (Merck) over 1 min with continuous stirring, followed by a further 1.5 min of continuous stirring. PEG was gradually diluted out using 2 mL RPMI-1640, 10 mM Glutamax added over 2 min, and a further 8 mL over an additional 2–3 min. Cells were left to incubate undisturbed for 20 min at RT. The fused cells were pelleted as before, and resuspended in fusion media. Cell density was adjusted to 1 × 10^6^ cells/mL and the cell suspension (100 µL/well), including unfused spleen cells as a control, was plated out into the above plates with mouse thymocytes as feeder cells (0.5–1.0 × 10^6^ cells/well) and cultured in the 37 °C, 5% CO_2_ incubator.

### Selection of positive hybridomas and cloning of monoclonal hybridomas

The cells were re-fed after 5 days growth by replacing 150 µL of media from each well with 200 µL of fusion media. After 10 days culture, an indirect ELISA was carried out to identify positive hybridomas specific to bovIP-10 by screening against the immunogen Fc-bovIP-10, recombinant bovIP-10 (Bio-Rad) and human IgG1-Fc tag (Roslin Toolbox) to exclude hybridomas that reacted against the Fc tag. All positive clones were switched to hybridoma HT media [complete media plus HT Supplement (Gibco, Thermo Fisher Scientific) and 6 ng/mL recombinant mouse IL-6] followed by expansion into 24 well plates, 6 well and T-25 tissue culture flasks upon reaching confluence. At this stage, the hybridomas represent multiclonal cell lines. These were cryopreserved in freezing media and stored in a −155 °C freezer.

The two best wells of multiclonal hybridomas, specific to bovIP-10 and with minimum binding to IgG-Fc, were single-cell sorted (BD FACS Aria III by the Bioimaging facility at RI) into 96 well plates with pre-grown thymocytes as feeder cells (0.5–1.0 × 10^6^ cells/well). Single-cell sorting was performed based on cells’ side scatter and forward scatter properties, serving as a faster and more precise alternative to serial dilution for high-throughput selection of monoclonal cells. The cells were cultured at 37 °C and 5% CO_2_, media replenished after 5–7 days. The supernatants from these single clones (which originated from single cell sorted hybridomas) were screened by ELISA on day 10 of culture. Cells from the wells that gave the highest ELISA readings i.e., specific reactivity to Fc-bovIP-10, and not against the Fc control, were passaged and grown up in 24-well plates. Upon confluence, the supernatants were confirmed for their bovIP-10 reactivity by an indirect ELISA using commercially available, purified IP-10, without the Fc label (Bio-Rad). The cells were also frozen at this stage, as described for the multiclonal stage, and mAb candidates were cultured for further expansion.

### Purification, isotyping and labelling of mAbs

Antibody was purified from centrifuged, filter-sterilised cell culture supernatant (harvested from five of the expanded clones) using a 1 mL HiTrap Protein G HP antibody purification column (Cytiva) as per manufacturer’s instructions. Briefly, samples were passed through the column by use of a peristaltic pump at a speed of 1.0 mL/min. Purified mAb was eluted using elution buffer (0.1 M Glycine buffer, pH2.7; Sigma-Aldrich) and neutralised with neutralising buffer (Tris–HCl, pH9.0; Sigma-Aldrich). Fractions containing purified mAb, as measured by Nanodrop spectrophotometer, were collected, pooled and buffer-exchanged to PBS using an Amicon® Ultra-4 Centrifugal Filter Unit with 50 kDa molecular weight cut-offs (MWCOs) (Merck). Concentrations of purified mAbs were measured using Nanodrop and these were stored at −20 °C. The purified mAbs were isotyped using IsoStrip™ Mouse Monoclonal Antibody Isotyping Kit (Merck), and aliquots were biotinylated using the One-Step Antibody Biotinylation Kit (Miltenyi Biotec, Surrey, UK) according to the manufacturer’s instructions. Antibodies were also labelled with an Alexa Fluor™ 647 (AF647) Antibody Labeling Kit (Molecular Probe; Thermo Fisher Scientific) as per the manufacturer’s instructions for use in intracellular staining. Similarly, isotype control mAb (purified mouse IgG1k, BioLegend) was labelled with AF647. The rest eight antibody-containing supernatants were concentrated 10- to 50-fold using Amicon® Ultra-15 centrifugal filter units (MWCO 50 kDa), following the manufacturer’s protocol.

### Antibody blocking assay

In order to develop a sandwich ELISA for detection of IP-10 using the newly generated mAb, it was essential to identify a pair of mAb that can be used for capture and detection. In order to do this, cross-recognition of IP-10 in a blocking assay was carried out: ELISA plates were coated with rbovIP-10 at 25 ng/well in 50 μL of Carbonate-Bicarbonate Buffer and placed overnight at 4 °C. The following day, the plates were washed with PBS-0.05% Tween-20 (PBST) thrice and blocked for 1 h using 0.1% casein in PBS-Tween-20 (100 µL per well). After washing, purified anti-bovIP-10 mAbs were incubated in duplicates for 1 h in 100-fold excess (25 μg in 0.1% casein) to enable complete binding to all epitopes on bovIP-10. The plates were washed and the subsequent binding of biotin-labelled anti-bovIP-10 mAbs (0.25 μg in 0.1% casein, 50 µL per well for 1 h) was determined in a checker-board fashion with Streptavidin–Horseradish Peroxidase conjugate (SAv-HRP, diluted 1 in 5000 with 0.1% casein, 50 µL per well for 45 min) (Thermo Fisher Scientific). Following a final three PBST washes, the next steps were performed as described above. Positive controls were the wells that only received biotin labelled mAbs only (i.e., no 100-fold excess purified mAbs were added and hence no blocking). Complete negative controls (buffer only) were also included. Percentage of inhibition was calculated relative to the positive control wells by the following formula, as described by other groups [23, 24].

$$\text{Percent inhibition}=\frac{ OD\, of\, positive\, controls-OD\, with\, blocking\, antibody }{OD\, of\, positive\, controls} \times 100$$

### Development and evaluation of 7C2 mAb based sandwich ELISA

All of the mAbs tested above were found to block each other and therefore could not be used as a pair (or pairs) for sandwich ELISA development. To enable further development, one mAb (7C2) was randomly selected and used as a capture antibody. This mAb was paired with a commercially available (Kingfisher Biotech, St Paul, MN, USA) pAb antibody used as a detection antibody. These were tested for detection of rBovIP-10 initially and compared with an ELISA using both capture and detection pAb from Kingfisher Biotech (as described by Goosen et al., [20]). The assay was used to detect recombinant bovIP-10 (Kingfisher Biotech). For the development of an assay with the 7C2 mAb, the following modifications were made to the protocol described by Goosen et al.: (i) the capture antibody (7C2) was used at a concentration of 10 µg/mL; (ii) the detection antibody (polyclonal anti-IP-10, Kingfisher Biotech, St Paul, MN, USA) was used at 0.2 µg/mL and (iii) a standard curve of recombinant bovine IP-10 (Kingfisher Biotech) ranging from 0 to 25 ng was used. Optical density (OD_450_-OD_550_) was measured as described above. The IP-10 ELISAs were compared using rbovIP-10 standard curves and quantification range of both assays determined as described above.

### Intracytoplasmic IP-10 staining

PBMCs were separated from heparinized whole blood based on gradient centrifugation using Lymphoprep (Serumwerk, Stemcell Technologies, Cambridge, UK) as previously described [25]. Frozen PBMCs from three individual calves were recovered from −150 °C freezer and washed with warm complete RPMI media [RPMI-1640 (Gibco, Thermo Fisher Scientific), 10% HI-FBS (Gibco, Thermo Fisher Scientific), 1 mM Glutamax, 100 U/mL penicillin and 100 µg/mL streptomycin (Gibco, Thermo Fisher Scientific)]. Viable cells were counted using a haemocytometer using trypan blue exclusion. Cells were seeded at 1.0 × 10^6^ cells/mL and stimulated with: either 5.0 µg/mL Concanavalin A (ConA) (Sigma-Aldrich), 2.5 µg/mL Pokeweed Mitogen (PWM) (Sigma-Aldrich) or left unstimulated. The chosen concentrations were based on prior work in our laboratory where strong cytokine responses were induced [26, 27]. After 20 h, cells were treated with BD GolgiStop™ (BD Biosciences, Becton Dickinson, Wokingham, UK) and cultured for a further 4 h. Cells were then harvested by centrifugation, washed and 2 × 10^6^ cells were added to wells of a 96 well plate. The cells were pelleted at 350 × *g* for 2 min and washed with PBS twice. Cells were then incubated in the dark with Zombie Yellow (1/1000) (BioLegend) at RT for 15 min. Cells were washed with PBS and resuspended with 100 µL per well of fixation/permeabilization solution (BD Biosciences) for 20 min at 4 °C. Cells were then washed twice in 1 × BD Perm/Wash™ buffer (BD Biosciences) and thoroughly resuspended with 50 µL of BD Perm/Wash™ buffer containing AF647 labelled 7C2 mAb and isotype control (mouse IgG1k-AF647, clone MOPC-21, BioLegend). Staining was carried out at 4 °C for 30 min in the dark. Then, cells were washed twice with 1 × BD Perm/Wash™ buffer and resuspended in Staining Buffer (5% normal goat serum (Abcam, Cambridge, UK) in PBS) prior to flow cytometric analysis with a BD LSRFortessa (BD Biosciences). A minimum of 50 000 cells were collected per sample. The flow cytometry results were analysed using FlowJo™ v10.8 Software (BD Life Sciences). Cells were first gated on Side Scatter Area (SSC-A) and Forward Scatter Area (FSC-A) to discriminate cell debris from the cell population. Dead cells were excluded by staining with Zombie Yellow, a viability dye that selectively penetrates cells with compromised membranes. Doublets and aggregates were discriminated based on signal processing by comparing Forward Scatter Area (FSC-A) and Forward Scatter Height (FSC-H). This ensured that only live and single cells were included in the subsequent analysis.

### Data analysis

ELISA data were analysed using Microsoft Excel (Microsoft Software, Redmond, WA). Percent inhibition was calculated as described above. The working range of the two ELISA assays were determined by calculating detection limit/sensitivity (i.e., lowest IP-10 level that can be quantified and discriminated from background) and upper limit of quantification (highest IP-10 level that can be quantified with reliability) as previously described [11, 28, 29]. Lower limit of detection was determined as the value (ng/mL) calculated by adding two times the standard deviation from a series of blanks to the mean OD value of blank wells and interpolated from the standard curve. For upper limit of quantification, three times the standard deviation of the highest point in straight part of the standard curve was subtracted from the mean and interpolated from the standard curve. The comparison between IP-10 levels in PPDb minus medium-stimulated WBS in *M. bovis* naïve and *M. bovis* positive groups was computed by Mann–Whitney U test. Flow data were analysed using FlowJo v10.8 (BD Life Sciences) above, and the groups compared using paired T test. Statistical analysis and plots were generated using GraphPad Prism version 9.0 for Windows (GraphPad Software, San Diego, CA, USA).

## Results

### Confirmation of expression of recombinant Fc-bovIP-10

It was essential to generate large quantities of recombinant bovIP-10 (rbovIP-10) for mouse immunisations to facilitate mAb production. Recombinant bovIP-10 was produced from a vector which enabled bovIP-10 to be expressed as a fusion construct with an Fc tag for enhanced immunogenicity and ease of subsequent purification. The predicted molecular weight by Expasy Compute pI/Mw tool [30] using protein sequence of Fc-bovIP-10 was 38 kDa. To confirm the molecular weight and identity, SDS-PAGE and mass spectrometry (MS) was performed. A band was detected at approximately 38 kDa by SDS-PAGE (Additional file 1, left panel). The sequence view of the MS results is shown in Additional file 1, (right panel). The blue bars show MS/MS spectra mapping to the peptide sequence. PEAKS studio software (Bioinformatics Solutions Inc.) analysis showed 86% sequence coverage for bovIP-10 and 34 out of 53 peptide sequences (−log_10_ p score 527) uniquely mapping to bovIP-10, confirming the identity.

### Mice responded specifically to immunisation with rbovIP-10

Standard hybridoma technology [18] was used to generate mAbs against bovIP-10. Mice (*n* = 3) were immunised with Fc tagged rbovIP-10 and their sera were tested by indirect ELISA to confirm the production of bovIP-10 specific antibodies. Screening was carried out against Fc-tagged rbovIP-10 or Fc control on day 1 (before immunisation) and on days 29 and 46 post-immunisation (Figure 1B). All three mice showed no reactivity against bovIP-10 or Fc control prior to immunisation. Post immunisation, by day 29, low levels of binding against both bovIP-10 and Fc control were detected in all 3 mice. By day 46, very strong binding responses against rbovIP-10 were detected from mice #1 and #2 but not for mouse #3. Since mouse #2 showed the highest binding against rbovIP-10, it was selected for further steps of mAb generation.

### Generation of bovIP-10 specific mAbs

**Figure 2:**
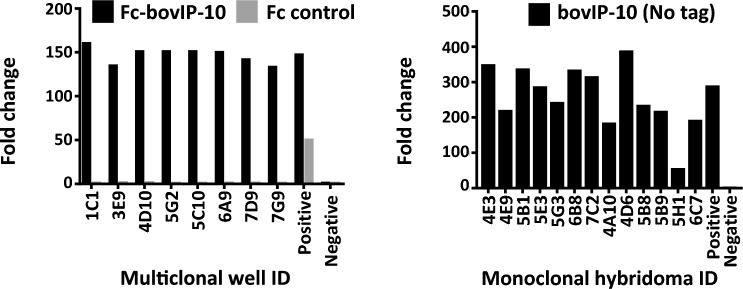
**Identification of hybridomas secreting bovine IP-10 specific antibodies**. ELISA screening of multiclonal hybridomas (left panel): plates were coated with Fc-bovIP-10 or Fc control protein. Eight multi-clonal candidates were identified specific to Fc-bovIP-10, with no reactivity against Fc. Two multiclonal hybridomas (5G2 and 7G9) were single cell sorted, cultured and re-screened to identify their specific reactivity against bovIP-10. Thirteen monoclonal hybridomas (right) were identified. Positive (sera from immunised mice diluted 1 in 1000) and negative (buffer only, secondary antibody only and media only) controls were included. Fold change refers to the enhanced bovIP-10 reactivity (measured by OD value) of hybridoma supernatants compared to the secondary antibody only control well.

The splenocytes from mouse #2 were fused Sp2/0-Ag14 cells and cultured in fusion media for 10 days. Neat supernatants (from multiclonal hybridomas at this stage) were screened for reactivity to Fc-bovIP-10 and Fc control by indirect ELISA. Positive (sera from immunised mice) and negative (buffer only, secondary antibody only and cell culture media only) controls were included. Eight multiclonal samples were shown to contain antibodies that were specific for Fc-bovIP-10, but no reactivity to the Fc control (Figure 2, left panel). Fold change refers to the enhanced Fc-bovIP-10 reactivity (measured by OD value) of hybridoma supernatants compared to the secondary antibody only control well. Based on their specific reactivity against Fc-bovIP10 and growth of the cells in culture, two multiclonal hybridomas i.e., 5G2 and 7G9 were taken forward and single cell sorting was performed. The single cells were cultured for 10 days, followed by indirect ELISA screening to identify wells/candidates specific for Fc-bovIP-10, and not against the Fc tag. Finally, 13 candidates were identified and their bovIP-10 specific reactivity was confirmed upon screening the neat supernatants against commercially available purified IP-10, without the Fc tag (Bio-Rad) (Figure 2, right panel). These clones were further cultured to generate monoclonal hybridoma cell lines. The first five mAb supernatants were selected for purification and isotyping – all of them were found to be mouse IgG1.

### All anti-bovIP-10mAs blocked each other

In order to develop an ELISA, a pair of mAb were needed. These mAbs would need to recognise different epitopes, leading to enhanced sensitivity of the assays. In order to determine suitability of mAb as pairs for ELISA, five mAbs were selected and biotinylated and cross-blocking was compared using biotinylated and non-biotin versions. A competition ELISA was set up to assess the inhibition of the binding of biotinylated mAbs by a 100-fold excess of the purified mAbs. Percentage inhibition was calculated and shown in Table 1. The results show that there was a very large degree of blocking, with each mAb blocking the ability of the other mAb to bind to IP-10. Biotinylated 7C2 was chosen to test the remaining eight mAbs in a blocking assay and also demonstrated a blocking effect (data not shown). This suggests that all mAbs blocked each other.

**Table 1:**
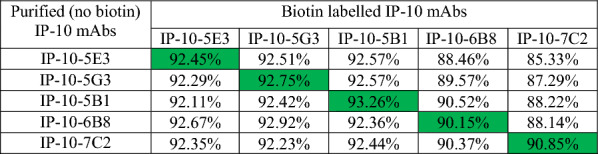
**Percent inhibition of the binding of biotinylated anti-bovIP-10 mAbs to rbovIP-10 by a 100-fold excess of the unlabelled (purified) anti-bovIP-10 mAbs**. Numbers in green show self-inhibition. Shown are inhibition percentages from one representative experiment (five experiments were performed).

### 7C2 mAb based ELISA showed an enhanced detection range

**Figure 3:**
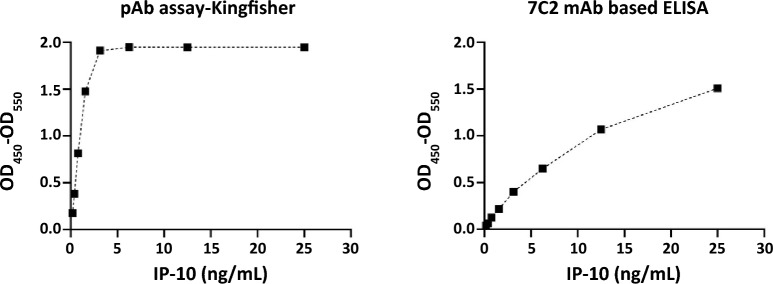
**Assessment of mAb 7C2 for detection of IP-10 by ELISA.** Commercial pAb based Kingfisher ELISA (left) and the assay incorporating 7C2 mAb (right) were tested with rbovIP-10 standard curve in triplicates. The y-axis shows the average optical density (OD_450_-OD_550_) values, the x-axis represents IP-10 concentration in ng/mL, and the black squares indicate points on the standard curve.

Since all of the tested mAbs recognised the same epitope of IP-10, it was not possible to select a pair for the development of a sandwich ELISA. As an alternative approach, mAb 7C2 was selected for further assessment as a capture mAb for ELISA. This was paired with a commercially available biotinylated pAb (Kingfisher Biotech) to develop a sandwich ELISA. As shown in Figure 3, the 7C2 mAb based ELISA, and the commercial Kingfisher ELISA enabled detection of rbovIP-10. The working range of both assays was determined by defining the detection limit/sensitivity (minimum IP-10 level that can be detected and discriminated from the background) and upper limit of quantification (maximum IP-10 level that can be reliably detected). The Kingfisher ELISA was found to be more sensitive i.e., 0.2 ng/mL compared to the assay using mAb 7C2 (sensitivity of 0.6 ng/mL). The 7C2 assay was found to be more sensitive than a commercial assay from Bio-Rad, which we have previously used [11]. Moreover, the 7C2 mAb based assay had greater linear range with accurate detection in the linear part of the standard curve of up to 5.4 ng/mL compared to the Kingfisher ELISA (3.6 ng/mL), although concentrations higher than this can be detected by both assays.

### 7C2 mAb ELISA detected IP-10 from *M. bovis* naïve and infected animal cohorts

**Figure 4:**
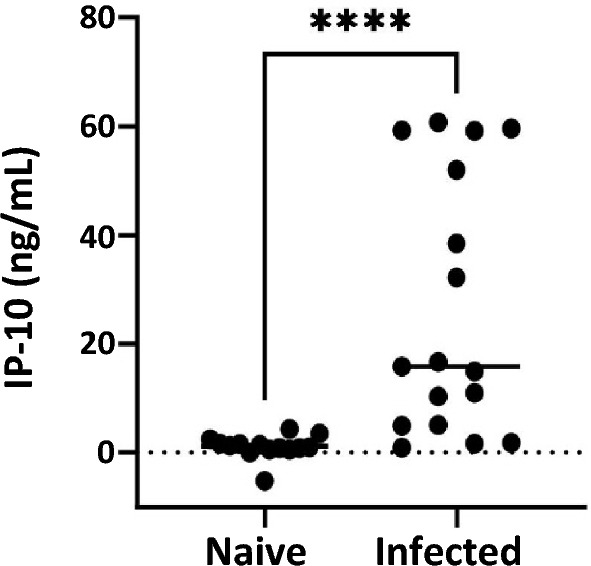
**Detection of antigen-specific IP-10 in bovine samples by ELISA.** The 7C2 mAb based ELISA was evaluated using samples from *M. bovis* naïve (*n* = 14) and culture positive (infected, *n* = 17) cohorts. Unstimulated (medium) and PPDb WBS samples were tested. PPDb minus medium levels of IP-10 are shown on y-axis for the two test groups. The results indicated a significantly elevated IP-10 in culture positive group. ^****^p < 0.001 (Mann Whitney U test.

The ELISA based on use of 7C2 as a capture mAb with a pAb detection antibody was utilised to measure IP-10 levels in samples obtained from field animal cohorts (Figure 4) to confirm that natural IP-10 can be detected. The samples tested were from *M. bovis* naïve animals (*n* = 14), and from cattle that were confirmed infected with *M. bovis* by culture (*n* = 17). Statistically significant higher levels of PPDb induced IP-10 were observed in the *M. bovis* infected animals (*p* < 0.0001) compared to the *M. bovis* naïve group (median group IP-10 concentrations of 15.8 ng/mL and 1.1 ng/mL respectively). This suggests that the 7C2 mAb based IP-10 ELISA assay is suitable for application in a clinically relevant sample matrix, and further confirms the differential potential of PPDb specific IP-10 for detection of *M. bovis* infection.

### Intracellular staining of bovine PBMC with AF647 labelled anti-bovIP-10 (7C2) mAb showed expression of IP-10 in response to mitogen stimulation

**Figure 5:**
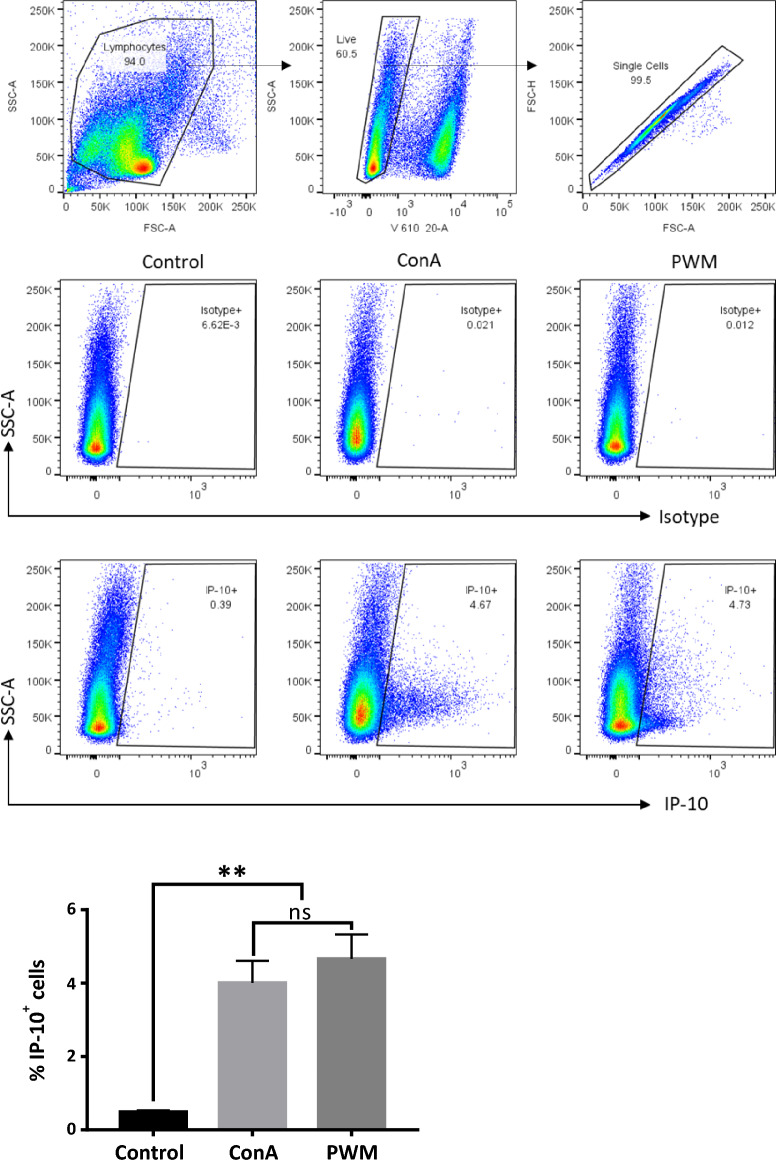
**Intracellular detection of bovine IP-10 in bovine PBMCs.** Frozen PBMCs were cultured overnight before stimulation with mitogens (ConA and PWM). Control cells were left unstimulated. GolgiStop protein transport inhibitor was added for the final 4 h. After Zombie yellow viability staining, cells were fixed and permeabilized before intracellular staining with AF647 labelled anti-bovIP-10 7C2 mAb. Live, single cells were gated (top panel) and analysed using FlowJo software. Middle panel from one representative experiment shows isotype control staining (mouse IgG1k-AF647) while the bottom panel shows IP-10 staining. The bar graph shows the percentage of IP-10^+^ cells in control and stimulated cells.

The ability of the 7C2 mAb to detect intracytoplasmic IP-10 was assessed. Bovine PBMCs were stimulated with ConA and PWM, and intracytoplasmic IP-10 was detected with AF647 labelled 7C2. Stimulation with either ConA or PWM induced significant expression of IP-10 by PBMCs. Figure 5 shows representative plots of intracellular IP-10 staining from one animal (top panel for isotype controls and bottom panel for IP-10 staining). On average 0.49 ± 0.04% of the control, unstimulated, PBMCs expressed intracellular IP-10 and the proportions increased to 4.01 ± 0.60% after ConA and to 4.66 ± 0.67% after PWM stimulation respectively.

## Discussion

The development of bovIP-10 mAbs was pursued in light of evidence that measurement of IP-10 can provide added diagnostic value when combined with IFN-γ for bTB detection. Being a major pro-inflammatory cytokine, IP-10 is involved in recruiting T cells to the site of inflammation i.e., the granuloma. Compared to control tissues, significantly upregulated IP-10 mRNA expression was reported in the granulomas of lymph node tissues of cattle experimentally infected with *M. bovis* [31]. Although a preliminary study did not support the potential of IP-10 protein levels as a biomarker for bTB [32]; Parsons et al., reported strong correlation between antigen specific release of IP-10 and IFN-γ, and PPDb stimulated IP-10 levels could differentiate skin test/IGRA positive and skin test/IGRA negative cattle with a sensitivity of 100% and specificity of 97% [33]. Additionally, determining IP-10 along with IFN-γ in plasma samples from blood stimulated with PPDb and defined TB antigens (ESAT-6, CFP-10, Rv3615c and TB7.7), was shown to identify additional confirmed TB positive animals in comparison to skin test and IGRA alone in cattle [14], and African buffaloes [13] respectively. Roles for IP-10 in other diseases such as mastitis [34] and paratuberculosis [35], have been reported, and this chemokine may also be altered by metabolic changes such as those associated with lactation in cattle [36]. The development of sensitive and specific detection platforms that could further determine the roles of IP-10 is urgently needed. An essential pre-requisite to achieve this is having a consistent access to homogenous, well characterized bovIP-10 mAbs; fuelling the rationale for this study. Novel mAbs for bovIP-10 detection were successfully developed and their use in ELISA and flow cytometric applications was explored.

All of the tested mAbs appeared to block each other. A potential explanation of this can be the small size of bovIP-10; resulting in a dominant B cell epitope and hence most generated mAbs showing a preferential epitope binding specificity. While self-inhibition among mAb candidates post hybridoma selection has been observed in other studies [23, 24, 37]; it is usual that mAb pairs can be identified enabling development of sandwich ELISAs. However, here it was not possible to use mAb pairs and further studies used a biotin labelled pAb alongside the mAb to develop a sandwich ELISA. Compared to polyclonal antisera, utilizing mAbs is reported to increase the overall accuracy, efficiency and specificity of ELISAs [38]. Although the 7C2 mAb based assay developed here was slightly less sensitive than the Kingfisher pAb based assay [14], it did show an enhanced linear range. This is important to allow simple quantification of bovIP-10 in a range of samples with varying levels of cytokine with high accuracy. In general, a broader quantification range offers benefits: more accuracy (as the extra dilution steps can be avoided); more applicability and reliability (testing samples where the concentrations can be unexpected, leading to fewer repeat assays) and a simplified analysis (in absence of a dilution factor to account for). Analysis of WBS from *M. bovis* infected and naive control animals using the 7C2 ELISA confirmed the previously reported IP-10 potential for bTB diagnosis [11–14] and demonstrated the suitability of the 7C2 mAb based ELISA in detecting native IP-10 in a diagnostically relevant matrix. The availability of a sensitive method to detect bovine IP-10 could enable studies of the role of IP-10 in health and disease.

Intracytoplasmic expression of IP-10 was detected using the 7C2 mAb: this was significantly enhanced following mitogen stimulation of bovine PBMCs compared to controls. The capability to measure intracellular IP-10 utilising this new mAb enables studies of specific cell subset-specific expression of this chemokine which may be important for defining correlates of immune protection, or how specific cell types influence disease progression or resolution.

Only one mAb (7C2) was studied here in detail. Future experiments could include more comprehensive characterization of this, and other mAb candidates, including cross reactivity testing for detection of IP-10 in other species. Further assessment of the mAb in LFAs would also be of benefit.

To conclude, the novel bovIP-10 mAbs panel and the applications described herein provide an opportunity and tools that be employed to delve deep into understanding of the kinetics of bovIP-10 in health and disease [28].

## Supplementary Information

**Additional file 1** **Confirmation of molecular weight and identity of Fc-bovIP-10***. *SDS-PAGE (A) showing a band at 38kDa for Fc-bovIP-10 (lane 10) and molecular weight marker (lane 2). MS (B): the blue lines depict MS/MS spectra mapping to the peptide sequence. PEAKS studio software analysis showed 86% sequence coverage for bovIP-10 and 34 out of 53 peptide sequences (-log_10_ p score 527) uniquely mapping to bovIP-10, confirming the identity.

## Data Availability

The datasets used and/or analysed during the current study are available from the corresponding author on reasonable request.
