# Supplementary figures and images for: Development and characterization of monoclonal antibodies specific for bovine IP-10
Source: Vet Res. 2025 Aug 14;56:169. doi: 10.1186/s13567-025-01602-z (PMC12351912; doi:10.1186/s13567-025-01602-z)

A


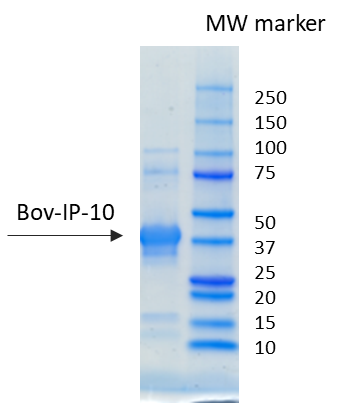


B


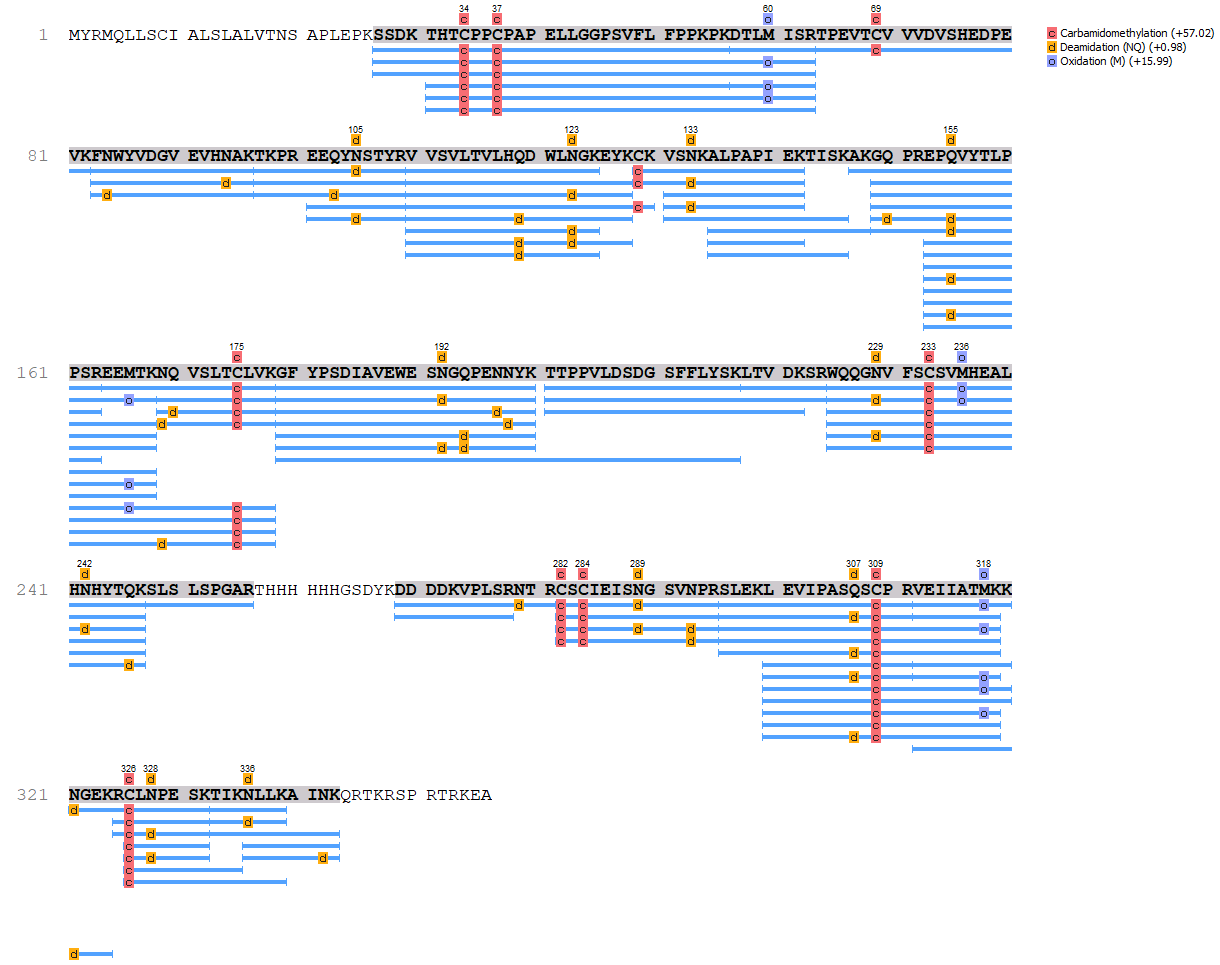

Supplement: Supplementary file 1 — Additional file 1 Confirmation of molecular weight and identity of Fc-bovIP-10. SDS-PAGE (A) showing a band at 38kDa for Fc-bovIP-10 (lane 10) and molecular weight marker (lane 2). MS (B): the blue lines depict MS/MS spectra mapping to the peptide sequence. PEAKS studio software analysis showed 86% sequence coverage for bovIP-10 and 34 out of 53 peptide sequences (-log10 p score 527) uniquely mapping to bovIP-10, confirming the identity. [file 13567_2025_1602_MOESM1_ESM.docx]
